# Synaptic Scaffolds, Ion Channels and Polyamines in Mouse Photoreceptor Synapses: Anatomy of a Signaling Complex

**DOI:** 10.3389/fncel.2021.667046

**Published:** 2021-07-28

**Authors:** Alejandro Vila, Eyad Shihabeddin, Zhijing Zhang, Abirami Santhanam, Christophe P. Ribelayga, John O’Brien

**Affiliations:** ^1^Richard S. Ruiz M.D. Department of Ophthalmology and Visual Science, University of Texas Health Science Center at Houston, Houston, TX, United States; ^2^MD Anderson Cancer Center, UTHealth Graduate School of Biomedical Sciences, Houston, TX, United States

**Keywords:** horizontal cell, KIR2.1, polyamine, Chapsyn110, feedback, rod, cone, rhythm

## Abstract

Synaptic signaling complexes are held together by scaffold proteins, each of which is selectively capable of interacting with a number of other proteins. In previous studies of rabbit retina, we found Synapse-Associated Protein-102 (SAP102) and Channel Associated Protein of Synapse-110 (Chapsyn110) selectively localized in the tips of horizontal cell processes at contacts with rod and cone photoreceptors, along with several interacting ion channels. We have examined the equivalent suites of proteins in mouse retina and found similarities and differences. In the mouse retina we identified Chapsyn110 as the scaffold selectively localized in the tips of horizontal cells contacting photoreceptors, with Sap102 more diffusely present. As in rabbit, the inward rectifier potassium channel Kir2.1 was present with Chapsyn110 on the tips of horizontal cell dendrites within photoreceptor invaginations, where it could provide a hyperpolarization-activated current that could contribute to ephaptic signaling in the photoreceptor synapses. Pannexin 1 and Pannexin 2, thought to play a role in ephaptic and/or pH mediated signaling, were present in the outer plexiform layer, but likely not in the horizontal cells. Polyamines regulate many ion channels and control the degree of rectification of Kir2.1 by imposing a voltage-dependent block. During the day polyamine immunolabeling was unexpectedly high in photoreceptor terminals compared to other areas of the retina. This content was significantly lower at night, when polyamine content was predominantly in Müller glia, indicating daily rhythms of polyamine content. Both rod and cone terminals displayed the same rhythm. While polyamine content was not prominent in horizontal cells, if polyamines are released, they may regulate the activity of Kir2.1 channels located in the tips of HCs. The rhythmic change in polyamine content of photoreceptor terminals suggests that a daily rhythm tunes the behavior of suites of ion channels within the photoreceptor synapses.

## Introduction

The visual world presents an enormous diversity of experience across intensity, spatial and temporal scales, presenting great challenges to extract useful information. Among the myriad mechanisms to enhance transmission of useful information is the establishment of antagonistic surround receptive fields that highlight the difference of the center signal from that detected nearby. At the first synapse in the visual system, that between the retinal photoreceptors and downstream bipolar and horizontal cells, an antagonistic surround is imposed on photoreceptor output (Thoreson and Mangel, 2012). The network of electrically coupled horizontal cells samples visual information from a wide area of retina and imposes feedback correlated to the average of the sampled area onto each individual photoreceptor synapse. This enhances contrast of local features and provides gain control at the photoreceptor output synapses.

The mechanisms of horizontal cell feedback to photoreceptors have been debated for decades. This is in part due to the fact that the “inverted polarity” of operation of vertebrate photoreceptors necessitates “inhibitory” signaling that defies traditional concepts of synaptic transmission. It is now widely accepted that the primary target of horizontal cell feedback is the photoreceptor voltage-gated calcium channel (Verweij et al., 1996; Kamermans and Spekreijse, 1999; Barnes et al., 2020), modulation of which alters the rate of photoreceptor transmitter release. Two mechanisms have emerged that have gained substantial experimental support: a proton-mediated mechanism in which modulation of the local pH within the photoreceptor synapse alters the degree of inhibition of the voltage-gated calcium channel (Hirasawa and Kaneko, 2003; Vessey et al., 2005; Vroman et al., 2014; Wang et al., 2014; Warren et al., 2016a; Grove et al., 2019), and an ephaptic mechanism in which ion channels in horizontal cell processes support electrical currents through the extracellular space of the invaginating synapse that create a voltage drop sensed by the photoreceptor voltage-gated calcium channel (Kamermans et al., 2001; Fahrenfort et al., 2005; Klaassen et al., 2011). Both cone and rod photoreceptors experience the same type of feedback (Thoreson et al., 2008; Babai and Thoreson, 2009), but the diversity of mechanisms reported from different groups of vertebrates and widely differing experimental measures of the weighting and even presence of the two core mechanisms in different species (Vroman et al., 2014; Warren et al., 2016b; Grove et al., 2019; Barnes et al., 2020) make it unclear how widely the mechanisms are conserved.

Both proton-mediated and ephaptic feedback mechanisms have characteristics that are enhanced by the unique structure of the deeply invaginated photoreceptor synapse complex. Localization of proteins that mediate some aspects of feedback mechanisms to this invaginating synapse is essential for the mechanisms to work. Indeed, localization of several proteins to horizontal cells processes within the invaginating synapses of photoreceptors has been taken as important evidence of the existence of proposed feedback mechanisms (Kamermans et al., 2001; Prochnow et al., 2009; Grove et al., 2019). In an earlier study, we have examined the distribution of MAGUK scaffold proteins that may anchor ion channels and other proteins in strategic locations that would allow them to be involved in horizontal cell feedback signaling in the rabbit retina (Vila et al., 2017). This study identified the scaffolds SAP102 and Chapsyn110 to be localized selectively in the tips of horizontal cell processes where they make invaginating contacts with rod and cone photoreceptors. Furthermore, the study identified known interacting partners of these scaffolds, including kainate receptor Glur6/7 and inward rectifier potassium channel Kir2.1, to be associated with this complex. The present study examines this suite of proteins in the mouse retina, finding the localization of a subset of these proteins to be conserved.

The inward rectifier potassium channel Kir2.1 generates a hyperpolarization-activated potassium conductance that, in principle, could provide an ephaptic feedback signal within the photoreceptor invaginating synapses (Vila et al., 2017). Rectification of inward rectifier potassium channels results from voltage-dependent channel block by intracellular polyamines (Williams, 1997; Baronas and Kurata, 2014). In this study, we also examine the distribution of intracellular polyamines and potential polyamine handling mechanisms. We find an unusually high concentration of polyamines unexpectedly in photoreceptor synaptic terminals, as well as a pronounced daily variation in this content.

## Materials and Methods

### Animals

Wild type (WT) C57BL/6J mice from Jackson Laboratory (Bar Harbor, ME; IMSR Cat# JAX:000664, IMSR_JAX:000664) were used for this study. All mice used were between 1.5 and 4 months of age; both male and female mice were used without preference. Mice were housed in a 12 h light/12 h dark cycle (lights on at 7:00 a.m.) for at least 2 weeks before an experiment. For experiments examining polyamine content of photoreceptors, some animals were maintained in a reversed light cycle for 2 weeks prior to experiments to facilitate collection of tissue in the night phase. Mice were sacrificed either 1 h before noon or 1 h past midnight, corresponding to daytime and nighttime, respectively. Day and night animals were used on different days. The day of the experiments, mice in the inverted cycle condition were kept inside black boxes until collection of retinal tissue was performed. The animals from both groups were sacrificed and tissue processed under infrared illumination with the assistance of night vision equipment as previously described (Li et al., 2013; Jin and Ribelayga, 2016). Mice were anesthetized by isoflurane inhalation and sacrificed by cervical dislocation. All procedures were approved by the Institutional Animal Care and Use Committee at the University of Texas Health Science Center at Houston and conform to National Institutes of Health (NIH) guidelines.

### Tissue Preparation and Immunohistochemistry

The eyes were enucleated, cut around the ora serrata, and the anterior segment and lens removed, resulting in a retina-sclera preparation. To prepare tissue sections, the retina-sclera preparation from the left eye was oriented by virtue of the insertion of the superior rectus muscle, cut in half on the nasal-temporal axis and the tissue pieces maintained in carboxygenated (95% O_2_ + 5% CO_2_) Ames’ medium (Sigma-Aldrich, St. Louis, MO) at ambient temperature. For light microscopy, the superior retina portion was immersion fixed in either 4% (w/v) paraformaldehyde (PFA) or 4% N-(3-dimethylaminopropyl)-N′-ethylcarbodiimide hydrochloride (EDAC; Sigma-Aldrich) in 0.1 M phosphate buffer (PB; pH 7.4). We found 30 min in 4% EDAC fixation followed by 10 min in 4% PFA was the best condition to visualize synaptic proteins. To visualize polyamine immunolabeling, the best condition was 4% PFA fixation for 45 min. Retinal pieces were cryoprotected in 30% sucrose in PB overnight at 4°C, embedded in OCT (Sakura Finetek, Torrance, CA), and sectioned vertically at 12 μm on a cryostat. For whole mount preparations, the right eye of each animal was dissected to make a retina-sclera preparation as above. Retinas were isolated from the retina-sclera preparation, flattened onto black nitrocellulose filter paper (EMD Millipore, Billerica, MA) and fixed in either 4% EDAC or 4% PFA for 1 h to preserve tissue integrity. The superior portion of the retina was used for imaging.

Retinal sections were treated with 0.3% Triton X-100 (Sigma-Aldrich) in Dulbecco’s Phosphate Buffered Saline (PBST), blocked with 3% Donkey Serum in PBST at room temperature (RT), and incubated overnight with primary antibodies in PBST + 3% Donkey Serum. For flat mount preparations, retinal pieces were incubated for a minimum of 5 days in a rotator at 4°C. Antibodies used included rabbit anti-SAP-102 (Thermo Fisher Scientific, Waltham, MA; AB_2546592), goat anti-SAP-102 (Abcam, Cambridge, MA; AB_777828), mouse anti-SAP-97 clone K64/15 (UC Davis/NIH NeuroMab Facility, Davis, CA; AB_2091920), mouse IgG1 anti-Chapsyn-110 clone N18/30 (UC Davis/NIH NeuroMab Facility; AB_2277296), mouse IgG2a anti-PSD-95 clone K28/43 (UC Davis/NIH NeuroMab Facility; AB_444362), mouse anti-CASK (Antibodies-online, Inc., Limerick, PA), sheep anti-mGluR6 (gift of Dr. Catherine Morgans; Morgans et al., 2007), mouse anti-GluR6/7 clone NL9 (Millipore-Sigma, Burlington, MA; Cat# 04-921, AB_1587072), rabbit anti-Kir2.1 (Alomone, Jerusalem, Israel; AB_2040107), guinea pig anti-Kir2.1 (Alomone; AB_2340970), rabbit anti-Connexin 57 (Invitrogen, Camarillo, CA; AB_2314266), rabbit anti-Pannexin 1 (Alomone; AB_2340917), rabbit anti-Pannexin 2 (Thermo Fisher Scioentific; AB_2533518), rabbit anti-Calbindin (Swant, Bellinzona, Switzerland; AB_1000034), mouse anti-Calbindin (Abcam; AB_1658451), mouse anti-Glutamine Synthetase (Millipore-Sigma; AB_2110656), and rabbit anti-SLC18B1 (Sigma Life Sciences, St. Louis, MO; AB_10600797). To visualize polyamines antibodies used included rabbit anti-Spermine (1:500; Novus, Littleton, CO; AB_10002326) and rabbit anti-Spermine (1:500, Abcam; AB_470871). Both antibodies recognize polyamine species including spermine, spermidine, and putrescine and their immunoreactivity was identical. The tissues were rinsed extensively in PBST following labeling. Additional information about the antibodies is given in Table 1 below.

**TABLE 1:** Antibodies used in this study.

Antibody	Host	Antigen	Source	Catalog # RRID	Dilution
SAP102	Rb	Recombinant fragment of human SAP102 (aa525–803)	Thermo Fisher Scientific, Waltham, MA, United States	PA5-29116 AB_2546592	1:500
SAP102	Gt	Synthetic peptide of human SAP102 (aa100–200)	Abcam, Cambridge, MA, United States	12086-200 AB_777828	1:250
PSD95	Ms	Fusion protein of human PSD95 (aa77–299)	UC Davis/NIH Neuromab, Davis, CA, United States	75-028 AB_2292909	1:500
SAP97	Ms	Fusion protein of rat SAP97 (aa1–104)	UC Davis/NIH Neuromab, Davis, CA	75-030 AB_2091920	1:250
Chapsyn 110	Ms	Fusion protein of rat Chapsyn 110 (aa1–852)	UC Davis/NIH Neuromab, Davis, CA	75-057 AB_2277296	1:500
CASK	Ms	Recombinant protein corresponding to aa318–415 of rat CASK	Antibodies-online Inc., Limerick, PA, United States	ABIN5774906	1:500
mGluR6	Sh	C-terminus of human mGluR6 (KATSTVAAPPKGEDAEAHK) coupled to KLH	Dr. Catherine Morgans, Oregon Health Sci U, Beaverton, OR, United States	N/A	1:500
GluR6/7	Ms	Rat GluR6 (aa894–908) coupled to KLH	Millipore, Temecula, CA, United States	04-921 AB_1587072	1:200
Kir2.1	Rb	Peptide NGVPESTSTDTPPDIDLHN from C-terminus of human Kir2.1 (aa392–410)	Alomone, Jerusalem, Israel	APC-026 AB_2040107	1:400
Kir2.1	GP	Peptide NGVPESTSTDTPPDIDLHN from C-terminus of human Kir2.1 (aa392–410)	Alomone, Jerusalem, Israel	AGP-044 AB_2340970	1:250
Pannexin 1	Rb	Peptide KEPTEPKFKGLRLE, corresponding to aa18–31 of human PANX1	Alomone, Jerusalem, Israel	ACC-234 AB_2340917	1:250
Pannexin 2	Rb	Synthetic peptide derived from C-terminus of mouse Pannexin 2	ThermoFisher, Waltham, MA, United States	42-2800 AB_2533518	1:200
Cx57	Rb	Synthetic peptide derived from mouse Cx57 (aa 434–446)	Invitrogen, Camarillo, CA, United States	40-4800 AB_2314266	1:100
Calbindin	Rb	Recombinant rat calbindin D-28k	Swant, Bellinzona, Switzerland	CB-38 AB_10000340	1:2,500
Calbindin	Ms	Synthetic peptide corresponding to bovine calbindin (clone CB-955)	Abcam, Cambridge, MA, United States	ab82812 AB_1658451	1:100
Glutamine synthetase	Ms	Glutamine synthetase purified from sheep brain	Millipore, Temecula, CA, United States	MAB302 AB_2110656	1:1,000
Spermine	Rb	Spermine conjugated to KLH	Novus, Littleton, CO, United States	NB100-1846, AB_10002326	1:500
Spermine	Rb	Spermine conjugated to KLH	Abcam, Cambridge, MA, United States	ab26975 AB_470871	1:500
SLC18B1	Rb	Recombinant protein epitope signature tag FYLLEYSRRKRSKSQNILSTEEERTTLLP	Sigma Life Science, St. Louis, MO, United States	HPA029747 AB_10600797	1:50

Most secondary antibodies were raised in donkeys and affinity purified. These included Alexa Fluor 488 and/or Cy3 anti-goat IgG (1:1,000; Jackson Immunoresearch, West Grove, PA), Alexa 488 and/or Cy3 anti-rabbit IgG (1:500; Molecular Probes, Eugene, OR) and DyLight 647 anti-mouse IgG (1:500; Jackson Immunoresearch). Additionally, anti-mouse IgG subtype specific secondary antibodies raised in goats were used at times to double label with two mouse monoclonals. These included Alexa 488 anti-mouse IgG1 and Cy3 anti-mouse IgG2a (1:500; Jackson Immunoresearch). Sections were incubated in secondary antibodies for 1 h at room temperature, and wholemount pieces of retina were left in secondary antibody for 1 day at 4°C, followed by extensive washes in PBST. Tissues were coverslipped in Vectashield mounting medium with DAPI (Vector Laboratories, Burlingame, CA).

### Confocal Microscopy

Image acquisition was performed with a Zeiss LSM 510 META or LSM 780 laser scanning confocal microscope (Carl Zeiss, Thornwood, NY). All sections were imaged with dye-appropriate filters (405 nm excitation, 440–460 nm emission for DAPI; 488 nm excitation, 530–550 nm emission for Alexa 488; 542 nm excitation, 590–620 nm emission for Cy3; 633 nm excitation, long-pass 650 nm emission for Dyelight 647). The detector gain and offset parameters were adjusted so that the intensity of most pixels fell within the dynamic range of the detector and the intensity of the most brightly labeled immunoreactive puncta within regions of interest to be examined showed very limited saturation. In some cases, large areas of labeling such as somata were saturated so that fine structures such as dendritic tips could be seen. Images were acquired with a 40× or 63× oil-immersion objectives as a series of optical sections ranging between 0.25 and 0.5 μm in step size. Each marker was assigned a pseudocolor and the images were analyzed as single optical sections and as stacks of optical sections projected along the y or *z*-axis. All images were processed in Adobe Photoshop (Adobe Systems CS5, San Jose, CA) to enhance brightness.

### Intensity Measurements of Polyamines at Different Times of the Day

To measure polyamine levels, 12 μm cryostat sections of superior retina were immunostained with rabbit anti-Spermine (1:500; Novus; AB_10002326) and mouse IgG2a anti-PSD-95 clone K28/43 (UC Davis/NIH NeuroMab Facility; AB_444362) antibodies. Five areas of the retina were scanned from at least three sections per animal; 4 animals were used for each experimental condition. 8-bit images from single confocal slices were analyzed with SimplePCI software (Hamamatsu Photonics, Bridgewater, NJ). The fluorescence intensity of Cy3 anti-spermine label was measured within circular regions of interest (ROIs) centered on photoreceptor terminals identified by labeling with anti-PSD95 antibodies. The mean intensity of polyamine labeling within ROIs was measured in both cones and rods, treating them as separate populations. In order to compare conditions, the average of polyamine labeling intensity from the photoreceptors of all 5 images was used to represent the polyamine labeling intensity in each animal. Identical acquisition settings were used to measure polyamine levels from animals in both conditions. Changes in average polyamine label intensity were evaluated between day and night conditions using two-way ANOVA with factor 1 being the time of day (day or night) and factor 2 being photoreceptor type (rods or cones), followed by Tukey’s multiple comparison tests.

### Analysis of Mouse Retina Single-Cell Transcriptome Data

Mouse retinal single-cell transcriptome data from Hoang et al. (2020) were accessed via the St. Jude Children’s Research Hospital ProteinPaint website.^1^ The data examined combine 11 datasets containing 1,702–5,966 cells each. 7 of these datasets derive from light-damaged retina, so the average gene expression reported includes both light-damaged and non-damaged retina. Individual genes of interest were queried and mean expression level data by cell type were captured. These data were replotted as heat maps using Prism software (GraphPad, San Diego, CA).

## Results

### Synaptic Scaffolds Occupy Distinct Niches in Photoreceptor Synapses

We have previously examined the distribution of synaptic scaffold proteins in rabbit photoreceptor synapses (Vila et al., 2017), finding that the membrane-associated guanylate kinase (MAGUK) scaffold proteins Synapse-Associated Protein 102 (SAP102 or Dlg3) and Channel Associated Protein of Synapse-110 (Chapsyn110 or Dlg2; also known as PSD93) assembled a complex of ion channels in the tips of horizontal cells. This complex is presumed to play a role in synaptic signaling between photoreceptors and horizontal cells. To have a better understanding of the conservation of this complex among species, we pursued a similar examination of synaptic scaffold proteins in the mouse retina.

**FIGURE 1:**
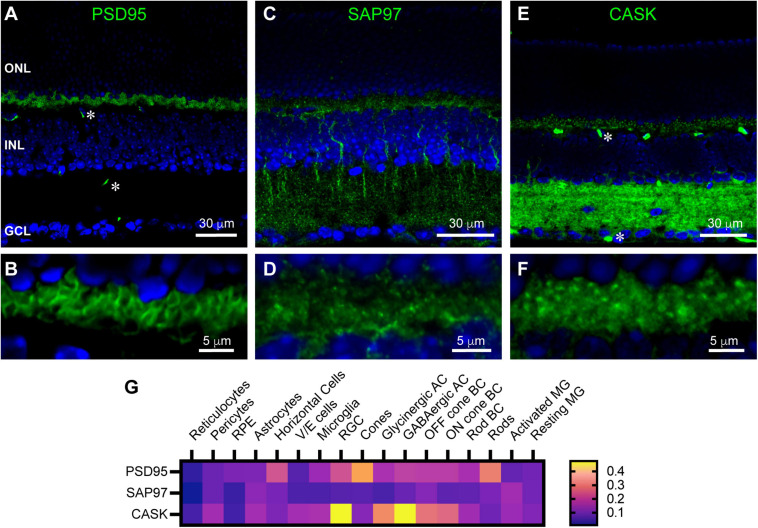
Localization of synaptic scaffolds PSD95, SAP97, and CASK in the mouse retina. **(A)** Immunostaining for PSD95 (green) with DAPI counterstaining (blue). Labels for retinal nuclear layers are: ONL, outer nuclear layer; INL, inner nuclear layer; GCL, ganglion cell layer. PSD95 labeling was prominent in the outer plexiform layer (OPL—between ONL and INL) and very weak in the inner plexiform layer (IPL—between INL and GCL). Two examples of non-specific labeling of blood vessels by the secondary antibody are indicated by asterisks. Two μm confocal stack. **(B)** Higher magnification view of the OPL showing PSD95 labeling of rod and cone terminals. Single confocal section. **(C)** SAP97 immunostaining revealed punctate and diffuse labeling in both OPL and IPL. Retinal layers are aligned as in **(A)**. One μm confocal stack. **(D)** Higher magnification view of the OPL shows SAP97 labeling to be diffusely distributed throughout the layer. One μm confocal stack. **(E)** CASK immunostaining revealed moderately strong labeling in the OPL and very strong labeling throughout the IPL. Retinal layers are aligned as in **(A)**. Two examples of non-specific labeling of blood vessels by the secondary antibody are indicated by asterisks. Two μm confocal stack. **(F)** Higher magnification view of the OPL shows CASK labeling to be diffusely distributed throughout the layer. Two μm confocal stack. **(G)** Average mRNA expression levels of PSD95 (Dlg4), SAP97 (Dlg1) and CASK (Cask) in mouse retina by single-cell RNA Sequencing. Of note in the OPL is some expression of PSD95 in horizontal cells, broad, low-level expression of SAP97, and expression of CASK most prominently in bipolar cells. Data adapted from Hoang et al. (2020). See section “Materials and Methods” for caveats about data interpretation. Abbreviations used: RPE, retinal pigmented epithelium; V/E cells, vascular endothelial cells; RGC, retinal ganglion cells; AC, amacrine cell; BC, bipolar cell; MG, Müller glia.

The MAGUK scaffolding protein Post-Synaptic Density Protein 95 (PSD95 or Dlg4) is well-known to have a pre-synaptic distribution in photoreceptor terminals in mammalian retina (Koulen, 1999; Li et al., 2013; Vila et al., 2017). In keeping with this, we observed PSD95 to clearly outline rod photoreceptor terminals in the outer plexiform layer (OPL) of mouse retina (Figures 1A,B). Little to no PSD95 was detectable in the inner plexiform layer (IPL). Two other synaptic scaffolds gave immunolabeling consistent with a pre-synaptic distribution in the OPL. Synapse-Associated Protein 97 (SAP97 or Dlg1) was previously seen to be widely distributed in both OPL and IPL of rat and rabbit retina (Koulen, 1999; Vila et al., 2017). In mouse, we found SAP97 immunoreactivity to be widespread and partially punctate in the IPL (Figure 1C), and rather diffuse and poorly delimited in the OPL (Figure 1D). SAP97 also labeled thick processes descending through the inner nuclear layer (INL), raising the possibility that this protein is also present in Müller glial cells. Finally, the MAGUK family member Calcium/Calmodulin-Dependent Serine Protein Kinase (CASK) was abundantly present throughout the IPL (Figure 1E) and present in dense blobs consistent with photoreceptor terminals in the OPL (Figure 1F). This is consistent with previous studies demonstrating its presence in photoreceptor terminals (Nuhn and Fuerst, 2014). Note that antibodies to PSD95 and CASK used were mouse antibodies, so labeling of blood vessels in Figures 1A,E (examples marked with asterisks) is a non-specific result of secondary antibody binding.

As an alternative strategy to investigate expression of these scaffolds in retinal cell types, we examined mouse retinal single-cell transcriptome data (Hoang et al., 2020), accessed through https://proteinpaint.stjude.org/F/2019.retina.scRNA.html. These data derive from light damage experiments and represent an average of data from undamaged, light damaged and recovering retina. In these datasets, PSD95 was present most abundantly in rod and cone photoreceptors, consistent with immunolabeling, but transcripts were also detected in horizontal cells and several types of bipolar cell (Figure 1G). Transcripts for SAP97 and CASK were also detected in a number of cell types contributing to the OPL. SAP97 was most abundant in Off bipolar cells, horizontal cells, rods and Müller glia, while CASK was most abundant in Off and On bipolar cells and Müller glia, with lower transcript levels in cones and rods. These data are partially consistent with the immunolabeling we found, and provide insight into why specific structures were difficult to resolve by immunostaining.

**FIGURE 2:**
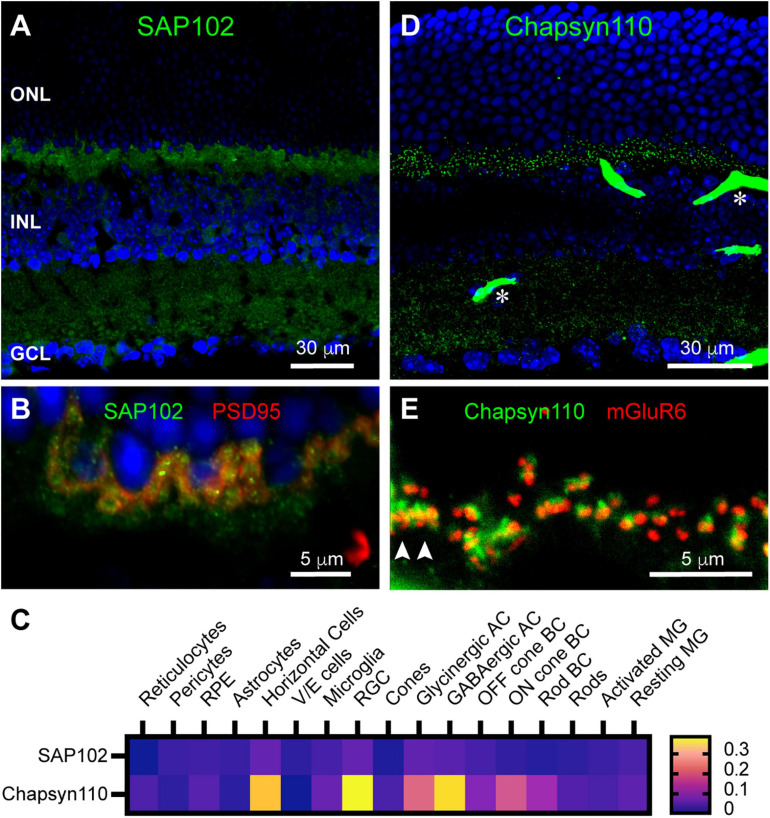
Localization of synaptic scaffolds SAP102 and Chapsyn110 in the mouse retina. **(A)** Immunostaining for SAP102 (green) with DAPI counterstaining (blue). Labels for retinal nuclear layers are as in Figure 1. SAP102 displayed partially punctate and partially diffuse labeling throughout both inner and outer plexiform layers. Five μm confocal stack. **(B)** Higher magnification view of the OPL labeled for SAP102 (green) and PSD95 (red). Two μm confocal stack. **(C)** Average mRNA expression levels of SAP102 (Dlg3) and Chapsyn110 (Dlg2) in mouse retina from single-cell transcriptome data. SAP102 displayed low levels of expression in horizontal cells and Müller glia, and lower levels in rods and cones. Chapsyn110 displayed prominent expression in horizontal cells and some expression in bipolar cells, particularly ON types. Data adapted from Hoang et al. (2020). **(D)** Immunostaining for Chapsyn110 revealed a sharply punctate distribution in both the outer and inner plexiform layers. Retinal layers are aligned as in **(A)**. Several prominent blood vessels non-specifically labeled by the secondary antibody are present, with two denoted by asterisks. Five μm confocal stack. **(E)** Higher magnification view of the OPL labeled for Chapsyn110 (green) and mGluR6 (red), labeling the tips of On bipolar cell dendrites. Chapsyn110 aligned closely with mGluR6, but did not co-localize with it. Arrowheads point to clusters of labeling for both markers at a cone pedicle. Three μm confocal stack.

We were particularly interested in the complex of proteins assembled in horizontal cell dendritic and axon terminal tips, so we also examined the distributions of SAP102 and Chapsyn110, previously found to anchor this complex in rabbit B-type horizontal cells (Vila et al., 2017). Unlike its restricted distribution in rabbit retina, SAP102 immunoreactivity was found diffusely in the mouse OPL (Figure 2A). SAP102 was also widespread and partially punctate in the IPL (Figure 2A), similar to the distribution of SAP97. At higher magnification it is evident that SAP102 was present within photoreceptor terminals labeled with anti-PSD95 (Figure 2B). However, additional labeling below the photoreceptor terminals in the OPL suggests that SAP102 was also diffusely present in horizontal cells, or perhaps Müller glial cells. Examination of single-cell transcriptome data (Figure 2C) revealed that SAP102 was expressed at quite low levels overall, but most prominently in horizontal cells, consistent with the prior rabbit retina results. Lower expression was noted in Müller glia, Off bipolar cells and rods. In contrast to the diffuse distribution of SAP102, Chapsyn110 immunoreactivity in mouse retina was sharply punctate in the OPL (Figure 2D). This distribution resembles that of metabotropic glutamate receptor mGluR6 at the tips of ON bipolar cell dendrites. However, Figure 2E shows that Chapsyn110 immunolabeling only weakly co-localized with that of mGluR6. Almost every ON bipolar cell dendritic tip labeled with mGluR6 was paired with a prominent spot of Chapsyn110, which was generally localized slightly above the mGluR6. This was true both for tight clusters of mGluR6 derived from ON cone bipolar cells contacting cone terminals (arrowheads) and for mGluR6 doublets derived from rod bipolar cells contacting rod spherules (most other signals in Figure 2E). Examination of single-cell transcriptome data (Figure 2C) revealed that Chapsyn110 was expressed at a high level in horizontal cells, and at a lower level in On cone and rod bipolar cells, consistent with the immunolabeling seen in the OPL.

### Chapsyn110 Is Associated With Horizontal Cell Dendritic and Axon Terminal Tips

**FIGURE 3:**
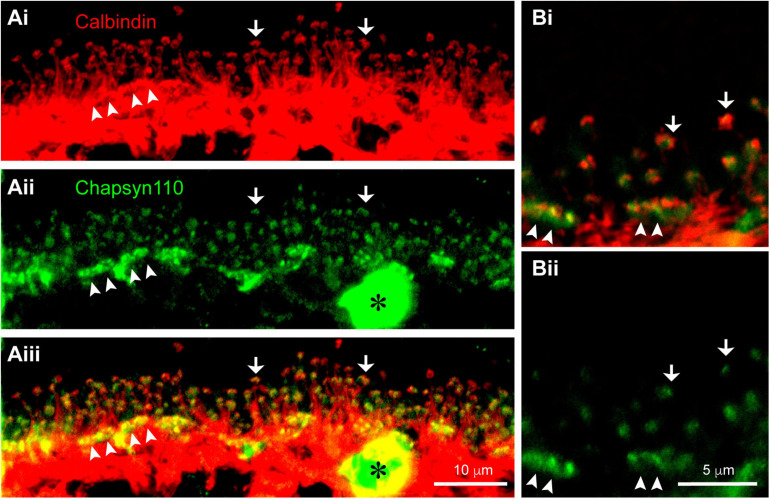
Association of scaffold Chapsyn110 with mouse horizontal cells. **(Ai)** Immunostaining for Calbindin (red) labels horizontal cells in the OPL. Fine processes rising up are axon terminal processes contacting a single rod at hook-like ending (arrows). Arrowheads point to clusters of dendritic processes contacting cone pedicles. Five μm confocal stack. **(Aii)** Chapsyn110 immunostaining (green) shows dense clusters of strong labeling (arrowheads) corresponding to locations of cone pedicles and small, isolated clusters of labeling corresponding to the tips of horizontal cell axon terminal processes (arrows). A blood vessel non-specifically labeled by the secondary antibody is marked with an asterisk. **(Aiii)** Merged view of Calbindin and Chapsyn110 labels reveals co-localization at tips of horizontal cell processes contacting both rods and cones. **(Bi)** Higher magnification view of horizontal cell processes contacting rods and cones. Labeling scheme is the same as in **(A)**. **(Bii)** Chapsyn110 labeling in isolation. Chapsyn110 clustered in direct association with horizontal cell process tips, but also in adjacent non-horizontal cell spaces, most likely representing tips of ON-type rod and cone bipolar cells. 0.5 μm confocal stack.

In the mouse retina, a unique type of axon-bearing horizontal cell, which is morphologically similar to B-type horizontal cells in the rabbit, sends dendritic processes contacting cone pedicles and axon terminal processes contacting rod spherules (Peichl and Gonzalez-Soriano, 1994). The non-overlapping, close association of Chapsyn110 immunoreactivity with that of mGluR6 strongly suggests that Chapsyn110 is located in the tips of the horizontal cell processes where they form the lateral elements of the synaptic complexes with photoreceptors. To examine this association, we labeled horizontal cells with antibodies to Calbindin (Figure 3Ai). This labeling displays the long, thin axon terminal extensions of horizontal cells that reach up to contact each rod spherule (arrows). In addition, the horizontal cells also contact cone pedicles in dense clusters of short dendritic extensions (arrowheads). Figure 3Aii shows that Chapsyn110 labeling closely followed the labeling of horizontal cells, with visible clusters in positions near the tips of the horizontal cell processes. The merged view (Figure 3Aiii) shows that Chapsyn110 labeling co-localized with the tips of the horizontal cell processes. Figure 3B shows a higher magnification view of this co-localization. Chapsyn110 labeling was particularly dense at the tips of dendrites contacting cone pedicles (arrowheads), although it was clearly present as well at axon terminal tips contacting rods (arrows). Note that the large spot of label in the Chapsyn110 channel (asterisk) is a blood vessel non-specifically labeled by the anti-mouse secondary antibody.

### Ion Channels Associated With Horizontal Cells

**FIGURE 4:**
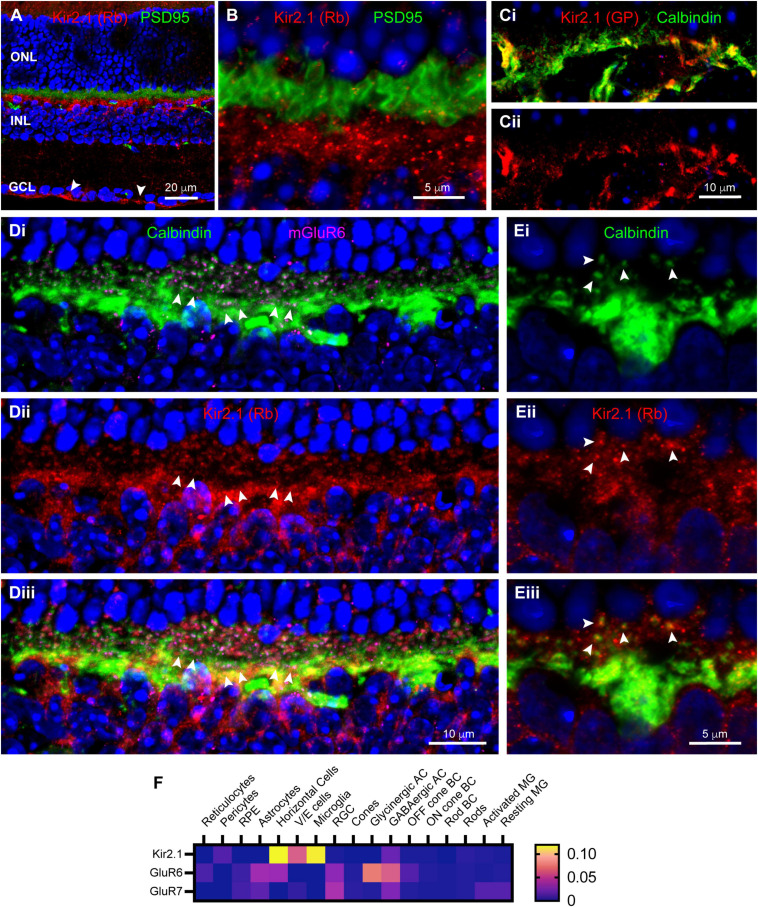
Localization of inward rectifier potassium channel Kir2.1 in mouse retina. **(A)** Immunostaining for Kir2.1 (rabbit antibody; red) with DAPI counterstaining (blue) and PSD95 (green) to label photoreceptor terminals. Labels for retinal nuclear layers are as in Figure 1. Kir2.1 is diffusely present in the IPL, but strongly labeled at the inner limiting membrane (arrowheads) and in the OPL. Five μm confocal stack. **(B)** Higher magnification view of the OPL reveals Kir2.1 (red) labeling largely below the photoreceptor terminals (green; PSD95 labeling), but with some diffuse and punctate labeling among the terminals. Two μm confocal stack. **(Ci)** Kir2.1 labeling (guinea pig antibody; red) colocalizes with Calbindin labeled horizontal cells (green) in the OPL. **(Cii)** Kir2.1 labeling in isolation. **(Di)** Another view of horizontal cells in the OPL (Calbindin labeling; green) along with mGluR6 labeling (magenta) to show locations of On bipolar cell dendritic tips. Clusters of mGluR6 label indicate cone terminals; three examples are indicated with paired arrowheads. Six μm confocal stack. **(Dii)** Kir2.1 labeling (rabbit antibody; red) in the same section. **(Diii)** Merged view of all three labels. Kir2.1 shows both diffuse and punctate labeling in the vicinity of horizontal cell axon terminal projections contacting rods, but little labeling near clusters of dendritic processes contacting cones. **(Ei)** Higher magnification view of a horizontal cell labeled with Calbindin antibody (green). Several representative axon terminal tips are highlighted with arrowheads. One μm confocal stack. **(Eii)** Kir2.1 labeling in the same section. **(Eiii)** Merged view of the two labels shows that tips of horizontal cell axon terminal processes contain punctate clusters of Kir2.1 labeling (arrowheads). **(F)** Average mRNA expression levels of Kir2.1 (Kcnj2) and kainate receptor subunits GluR6 (Grik2) and GluR7 (Grik3) in mouse retina from single-cell transcriptome data. Kir2.1 is most prominently expressed in horizontal cells and essentially absent from Müller glia. The kainate receptor subunit GluR6 is also found in horizontal cells and Off bipolar cells, but GluR7 is absent from these cell types. Data adapted from Hoang et al. (2020).

Synaptic scaffolds anchor many proteins, including ion channels, in locations necessary for their functions. In a previous study in rabbit retina (Vila et al., 2017), we had identified the inward rectifier potassium channel Kir2.1, a known binding partner of Chapsyn110 and SAP102 (Leonoudakis et al., 2004; Leyland and Dart, 2004), as a partner in the complex of proteins at horizontal cell dendritic tips. We examined Kir2.1 distribution in mouse retina. Figures 4A,B show that immunolabeling with a rabbit antibody to Kir2.1 was particularly abundant in the OPL, below the level of photoreceptor terminals labeled for PSD95. Labeling was diffuse around the somas of horizontal cells, but also included clusters of puncta in the OPL. Kir2.1 labeling was sparser and more punctate in the outermost portion of the OPL. Labeling for Kir2.1 was also evident at the inner limiting membrane (Figure 4A, arrowheads), indicative of some expression in Müller glial cells. Because labeling with the rabbit anti-Kir2.1 antibody was somewhat ill-defined, we also examined labeling with a guinea pig anti-Kir2.1 antibody. The guinea pig antibody revealed the same overall distribution (not shown). Double-labeling with anti-Calbindin antibodies to label horizontal cells revealed that a substantial portion of the Kir2.1 labeling in the OPL was associated with horizontal cell somas and processes (Figure 4C). The guinea pig antibody was not particularly sensitive and did not reveal the sparse, punctate labeling in the outer OPL seen with the rabbit antibody. So, to further examine the association of Kir2.1 with horizontal cells, we labeled sections with the rabbit anti-Kir2.1 antibody and anti-Calbindin antibodies (Figure 4D). Antibodies to mGluR6 were also included to reveal positions of On bipolar cell dendritic contacts with cone and rod terminals (Figures 4Di,iii). Tight clusters of small mGluR6 puncta indicate cone terminals (three examples indicated with paired arrowheads), with closely associated horizontal cell dendritic processes (Figure 4Di). Kir2.1 labeling was very weak at the tips of these horizontal cell dendritic processes that contact cones. Kir2.1 labeling was more evident in the vicinity of the numerous horizontal cell axon terminal processes contacting rod spherules (Figures 4D,E). Most horizontal cell axon terminal tips contacting rod spherules displayed a punctate spot of Kir2.1 labeling (Figure 4E, arrowheads), although additional diffuse labeling spread beyond horizontal cell processes and may be associated with another cell, such as Müller glial cells. This distribution is less clearly defined than the distinct punctate clusters of Kir2.1 on horizontal cell dendritic and axon terminal tips previously detected in the rabbit retina (Vila et al., 2017). Analysis of single-cell transcriptome data (Figure 4F) confirmed prominent expression of Kir2.1 in horizontal cells, but revealed essentially none in Müller glia, inconsistent with our immunostaining results.

In our previous study in rabbit retina (Vila et al., 2017), we had also identified kainate receptors labeled with antibodies to GluR6/7 selectively localized at the tips of horizontal cell processes. We attempted to label mouse retina with these antibodies, but did not detect any labeling (data not shown). Examination of single-cell transcriptome data (Figure 4F) did reveal the presence of transcripts for GluR6 in horizontal cells, but not GluR7. This provides some support for the presence of GluR6 kainate receptors in horizontal cells, although we cannot provide insight into their localization.

**FIGURE 5:**
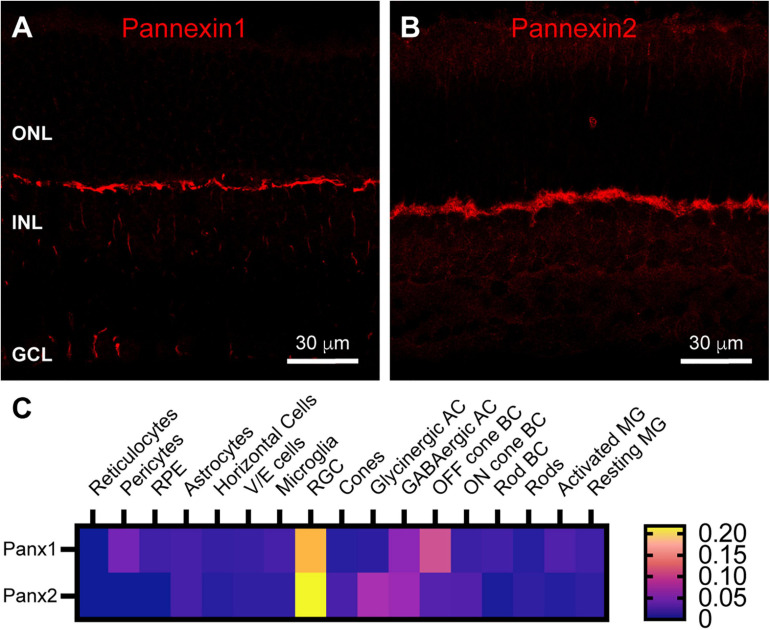
Pannexin labeling in mouse retina. **(A)** Immunostaining for Pannexin 1 in mouse retina revealed strong labeling in the outer plexiform layer and weaker labeling in the inner nuclear layer and ganglion cell layer. Three μm confocal stack. Labels for retinal nuclear layers are as in Figure 1. **(B)** Immunostaining for Pannexin 2 displayed strong labeling in the OPL and weaker labeling in the INL and photoreceptor layer. Three μm confocal stack. **(C)** Average mRNA expression levels of Pannexin1 and Pannexin2 in mouse retina from single-cell transcriptome data. In the OPL, the most prominent expression of Pannexin1 is in Off cone bipolar cells, with very weak expression in horizontal cells. Pannexin2 expression is largely absent from horizontal cells, but weakly present in On and Off bipolar cells. Data adapted from Hoang et al. (2020).

Horizontal cell feedback to cones is a complex process that may involve ephaptic communication and localized changes in pH within the photoreceptor synaptic cleft (Kamermans and Fahrenfort, 2004; Thoreson and Mangel, 2012; Vroman et al., 2014; Wang et al., 2014; Barnes et al., 2020). One type of channel thought to support ephaptic currents and ATP release to modulate pH is pannexin channels (Prochnow et al., 2009; Cenedese et al., 2017). We found strong labeling in the OPL for both Pannexin 1 and Pannexin 2 (Figures 5A,B). This is consistent with a previous finding of Pannexin 1 expression at some of the horizontal cell lateral elements of the photoreceptor triad synapse, as well as some bipolar cell dendrites in mouse retina (Kranz et al., 2013). Single-cell transcriptome data (Figure 5C) revealed only minimal expression of Pannexin 1 in horizontal cells and no Pannexin 2. Instead Pannexin 1 was most strongly expressed in Off bipolar cells in the OPL and in retinal ganglion cells. This pattern is consistent with our immunostaining (Figure 5A), which included labeling in ganglion cell somas. Pannexin 2 was also expressed strongly in retinal ganglion cells and to a lesser extent in amacrine cells, neither of which is consistent with our immunostaining (Figure 5B). The discord between Pannexin 2 immunostaining and transcriptome data make it difficult to draw conclusions about presence of this protein in OPL compartments.

Gap junction hemichannels comprised of connexins have been identified in horizontal cell processes contacting photoreceptors in fish and turtles (Kamermans et al., 2001; Pottek et al., 2003; Shields et al., 2007) and shown to play a role in ephaptic feedback (Klaassen et al., 2011). In rabbit retina, we previously found Cx57 and Cx59 to be present in horizontal cell gap junctions, but not to be detectable in the scaffolded complex at the tips of horizontal cell processes (Vila et al., 2017). We examined the distribution of Cx57 in mouse retina. Unfortunately, the antibodies we used did not produce labeling in mouse retina, so we were not able to assess the presence of hemichannels in horizontal cell processes.

### Daily Rhythms of Polyamine Content in the OPL

**FIGURE 6:**
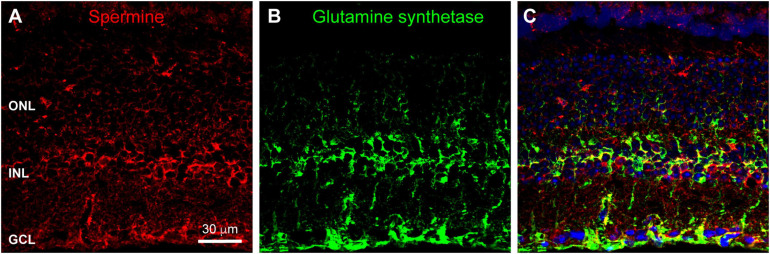
Polyamine labeling in the mouse retina. **(A)** Labeling for polyamines, as detected by an anti-Spermine antibody (red), was spread throughout all retinal layers. Labels for retinal nuclear layers are as in Figure 1. Eleven μm confocal stack. **(B)** Immunostaining for Glutamine Synthetase (green) labels Müller glia. **(C)** Merged view of the two labels shows that a substantial portion of polyamine labeling is co-localized with Müller glia.

**FIGURE 7:**
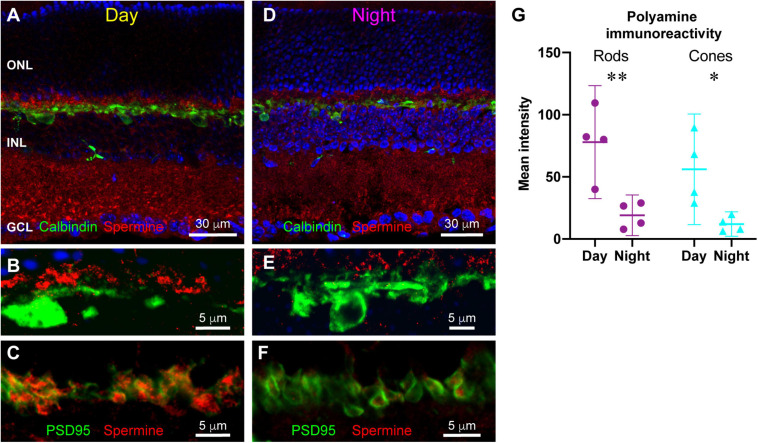
Differences in polyamine distribution between daytime and nighttime mouse retina. **(A)** Double labeling for polyamines (red) and Calbindin to label horizontal cells (green) in a retina sampled in photopic light 5 h after light onset (Day). Polyamine labeling is particularly prominent in the OPL. Five μm confocal stack. **(B)** Higher magnification view of the OPL in daytime retina. Prominent polyamine labeling is just above the horizontal cells (green; Calbindin label). Five μm confocal stack. **(C)** Double labeling for polyamines (red) and PSD95 (green) reveals that polyamines are located within photoreceptor terminals. Single confocal section. **(D)** Double labeling for polyamines (red) and Calbindin to label horizontal cells (green) in a retina sampled in darkness 5 h after light offset (Night). Polyamine labeling is overall less intense with distinctly less labeling in the OPL. Seven μm confocal stack. **(E)** Higher magnification view of the OPL in nighttime retina. Polyamine labeling (red) above horizontal cells (green) is far less prominent than in the daytime. Five μm confocal stack. **(F)** Double labeling for polyamines (red) and PSD95 (green) reveals that the reduced polyamine labeling in the nighttime OPL is still contained within photoreceptor terminals. Single confocal section. **(G)** Quantitative assessment of average polyamine labeling intensity in rod and cone terminals in day and night conditions. Points represent mean labeling intensity for all terminals measured for an individual animal (see section “Materials and Methods”). *p < 0.05, **p < 0.01, 2-way ANOVA with Tukey’s multiple comparisons.

Many channels are regulated by polyamine binding. Inward rectifier potassium channels acquire their rectification through voltage-dependent polyamine block of the channel: channels are blocked when the cell is depolarized and become unblocked and allow current flow when the cell hyperpolarizes (Baronas and Kurata, 2014). We hypothesized that polyamine content could regulate the activity of horizontal cell Kir2.1 channels and other ion channels in the OPL synaptic complex. To examine polyamine content, we labeled retina with antibodies to spermine, which detect various polyamine species including spermine, spermidine, and putrescine. Figure 6A shows that polyamine immunolabeling was widespread in the mouse retina. A substantial portion of the labeling coincided with the locations of Müller glia (Figures 6B,C), consistent with the relatively high accumulation of polyamines in retinal glia (Skatchkov et al., 2000). However, some labeling did not coincide with Müller cells, indicating polyamine presence in neurons, as has also been reported previously (Valentino et al., 1996). Double labeling with antibodies to Calbindin to label horizontal cells revealed at best a modest amount of polyamine content in the horizontal cells (Figures 7A,B). However, in retina collected in the daytime, there was strong polyamine labeling in the OPL above horizontal cells, suggestive of photoreceptor terminals (Figures 7A,B). Labeling with PSD95 to outline photoreceptor terminals revealed that this prominent polyamine content was indeed located within photoreceptor terminals (Figure 7C).

In retina collected in nighttime, polyamine labeling differed strikingly, being lower overall and showing less apparent concentration in the OPL in photoreceptor terminals (Figures 7D–F). To examine this difference quantitatively, we measured fluorescence intensity of spermine immunoreactivity in regions of interest within photoreceptor terminals identified by PSD95 labeling (Figure 7G) (see section “Materials and Methods” for details). In rod terminals, polyamine immunoreactivity was significantly higher in the daytime than at night (2-way ANOVA with Tukey’s multiple comparisons: mean difference 58.8; 95% confidence interval of difference 15.0–102.7; *p* = 0.0085; *n* = 4 animals in each condition). Cone terminals measured in the same images displayed the same effect (2-way ANOVA with Tukey’s multiple comparisons: mean difference 44.1; 95% CI of difference 0.3–88.0; *p* = 0.0482; *n* = 4 animals in each condition). There was no significant difference between rods and cones in either daytime or nighttime conditions. Thus, photoreceptor terminals displayed a strong daily rhythm of polyamine content, with much higher concentration present in the daytime than at night.

**FIGURE 8:**
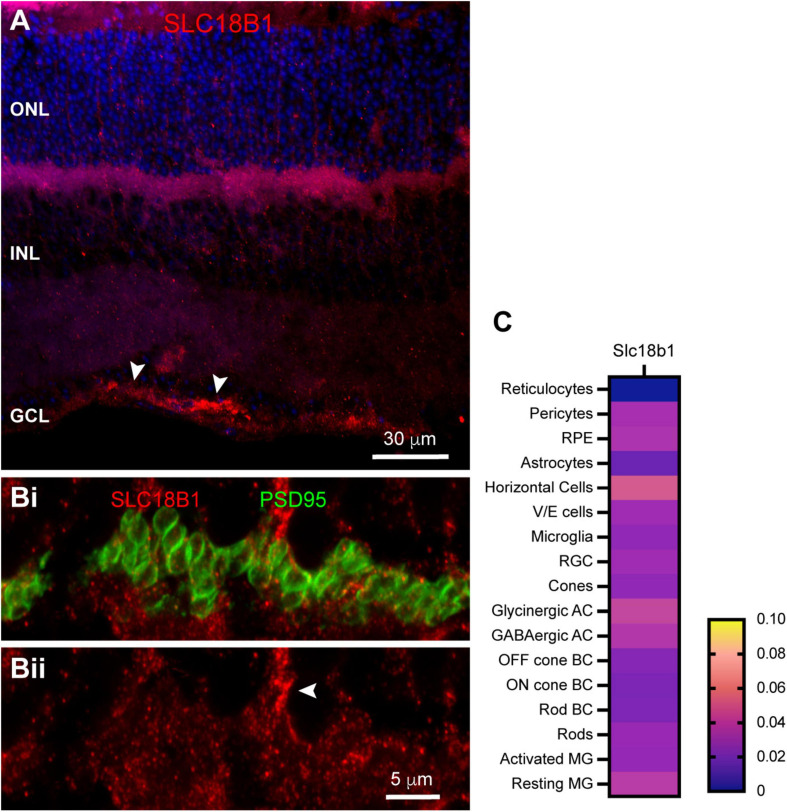
Localization of vesicular polyamine transporter SLC18B1 in mouse retina. **(A)** SLC18B1 immunostaining (red) with DAPI counterstain (blue). Labels for retinal nuclear layers are as in Figure 1. SLC18B1 was present diffusely through most of the retina, with more prominent labeling in the OPL and at the inner limiting membrane (arrowheads). Four μm confocal stack. **(Bi)** Higher magnification view of the OPL double labeled for Slc18B1 (red) and PSD95 (green) to label photoreceptor terminals. Eight μm confocal stack. **(Bii)** SLC18B1 labeling in isolation. Labeling is diffuse throughout the area of photoreceptor terminals and below them. Some areas of dense labeling (arrowhead) resemble Müller glial cell processes ascending through the ONL. **(C)** Average mRNA expression level of SLC18B1 in mouse retina from single-cell transcriptome data. SLC18B1 is present at very low levels in most retinal cell types, with horizontal cells and Müller glia showing somewhat higher levels. Rods also contained SLC18B1 transcripts. Data adapted from Hoang et al. (2020).

The high polyamine content in photoreceptor terminals led us to question whether polyamines could be released from photoreceptors into the extracellular space, where they might either regulate ion channels locally or be taken up into neighboring cells where they may regulate channels from the intracellular space. Polyamine packaging in synaptic vesicles and release in the brain has been recognized for some time (Masuko et al., 2003). Recently, the orphan transport protein SLC18B1 has been identified as a vesicular polyamine transporter (Hiasa et al., 2014). We labeled mouse retina sections with antibodies to SLC18B1. Labeling for SLC18B1 was evident primarily in the OPL and near the inner limiting membrane (Figure 8A). To evaluate whether SLC18B1 labeling in the OPL was associated with photoreceptors, we double-labeled with antibodies to PSD95 to outline photoreceptor terminals. Figure 8B shows that SLC18B1 was not specifically localized to photoreceptor terminals, but rather was spread throughout the OPL both above and below the terminals. Labeling was evident in some stout processes ascending into the ONL (Figure 8B, arrowhead), suggestive of Müller glial cells. This would be consistent with labeling near the inner limiting membrane (Figure 8A, arrowheads), which includes the Müller cell endfeet. In the single-cell transcriptome data, SLC18B1 mRNA was present at very low levels in many cell types throughout the retina (Figure 8C). Rods contained a modest amount of the transcript, but higher levels were detected in horizontal cells and Müller glia. Thus, it is feasible that polyamines found in photoreceptor terminals could be packaged into vesicles, but a more prominent role for the vesicular polyamine transporter may occur in the Müller cells, which also harbor some of the highest polyamine labeling (Figure 6).

## Discussion

Synaptic scaffolds bind a variety of proteins, assembling suites of proteins together, anchoring them in particular locations and coordinating synaptic functions (Montgomery et al., 2004; Oliva et al., 2012). We previously identified a synaptic scaffold complex in the rabbit retina that displayed a highly restricted distribution in the dendritic and axon terminal tips of B-type horizontal cells, where they form invaginated contacts with cone and rod photoreceptor terminals, respectively (Vila et al., 2017). By inference of the location of these complexes and the inclusion of selectively active channels such as kainate-type glutamate receptors and inward rectifier potassium channels, the complexes were hypothesized to contribute to synaptic signaling within the photoreceptor synapses. The present study was undertaken to assess the conservation of the suite of proteins that forms this synaptic complex in the mouse, in which the availability of genetic tools would facilitate further investigation of these hypotheses. This study confirmed the presence of key members of the suite of proteins, but also differences from the suite detected in the rabbit retina.

When compared to the rabbit retina (Vila et al., 2017), distributions of the MAGUK scaffolds PSD95 and Chapsyn110 in mouse retina were largely the same. In the outer plexiform layer, these two scaffolds had very clear distributions presynaptically in photoreceptor terminals and postsynaptically in the tips of horizontal cell processes, respectively. This suggests that certain proteins anchored by these scaffolds play conserved roles in signaling around the photoreceptor synapse. While none of the proteins we examined had distributions similar to PSD95, the plasma membrane calcium ATPases (predominantly PMCA1 and PMCA4) are distributed in precisely the same pattern in the OPL (Morgans et al., 1998; Krizaj et al., 2002; Haverkamp et al., 2003; Johnson et al., 2007), and PMCA4 is known to bind to PSD95 (DeMarco and Strehler, 2001). Thus, the critical role of calcium extrusion from the photoreceptor terminals is likely to be facilitated by this presynaptic scaffolded complex.

On the postsynaptic side, the restricted distribution of Chapsyn110 in small clusters at the tips of horizontal cell processes that invaginate into cone and rod photoreceptor terminals, conserved in both mouse and rabbit, suggests that the complex that it assembles plays a conserved role. Our previous work in rabbit retina identified two ion channels associated with this scaffold, inward rectifier potassium channel Kir2.1 and a kainate-type glutamate receptor labeled with antibodies against GluR6/7 (Vila et al., 2017). Antibodies to GluR6/7 that we used did not work in mouse (data not shown), so we were not able to assess whether this glutamate receptor was present in the tips of horizontal cell processes. However, conditional knockout of AMPA receptor GluA4 in mouse horizontal cells revealed a small remaining kainate receptor current (Stroh et al., 2013), indicating that a kainate receptor is present. Furthermore, kainate receptors labeled with antibodies to GluR6/7 have been observed in tips of horizontal cell processes in rat (Brandstatter et al., 1997), cat (Vardi et al., 1998), and macaque (Haverkamp et al., 2000; 2001), suggesting a conserved organization. Finally, kainate receptor subunit GluR6 was found in horizontal cells in mouse retina single-cell transcriptome data (Figure 4F; Hoang et al., 2020), suggesting that this organization is conserved in the mouse as well. On the other hand, Kir2.1 was clearly present in small clusters at the tips of the horizontal cell processes, as it was in the rabbit. Kir2.1 is known to bind to Chapsyn110 (Leyland and Dart, 2004), further suggesting that the presence of this scaffold in this restricted location may cluster Kir2.1 channels there. While we did not examine it, it is noteworthy that the GABA_C_ receptor ρ2 subunit also displays a restricted distribution in the tips of horizontal cell processes in mouse retina, comparable to that of Chapsyn110 (Grove et al., 2019; see also Haverkamp and Wassle, 2000). This GABA receptor has been proposed to play a central role in pH-mediated feedback to photoreceptors in mammalian retina (Grove et al., 2019; Barnes et al., 2020). Direct interactions between Chapsyn110 and GABA receptor subunits have not been reported. Finally, pannexins have also been proposed to play central roles in horizontal cell feedback signaling to photoreceptors in fish retina by releasing ATP into the synaptic cleft (Vroman et al., 2014; Cenedese et al., 2017). Pannexin1 has been identified in mouse horizontal cells and its knockout results in a small increase in ERG b-wave amplitude, possibly consistent with reduction of a feedback signal (Kranz et al., 2013). Our immunostaining results provide support for the presence of both Pannexin1 and Pannexin2 in OPL processes. However, mouse retina single-cell transcriptome data we examined (Figure 5C; Hoang et al., 2020) do not support their expression in horizontal cells, but rather in Off cone bipolar cells. Thus, it is unclear that pannexins contribute to the signaling within horizontal cell processes in the mouse retina.

In the rabbit retina, SAP102 also displayed a restricted postsynaptic distribution in the tips of B-type horizontal cell processes, but in mouse retina its distribution in the OPL was more diffuse, including presynaptic labeling in photoreceptor terminals and without clear clusters in horizontal cell processes. This suggests that the suites of proteins assembled at the photoreceptor synapses are likely to differ to some extent, but we have not yet performed an adequately extensive survey to determine how these complexes differ.

Perhaps the most novel finding of this study was the presence of a strong daily variation in polyamine content in rod and cone photoreceptor terminals. Polyamines have been detected chemically (Ientile et al., 1986; Withrow et al., 2002) and histologically (Valentino et al., 1996; Skatchkov et al., 2000; Withrow et al., 2002) in a number of cell types in the retina, including photoreceptors, ganglion cells, amacrine cells and Müller glia. Polyamine content in several cell types has been found to vary over the course of retinal development in rabbit (Withrow et al., 2002), declining somewhat in adults. Rather significantly, liver cellular polyamine content varies with time of day and can directly interact with core circadian clock components (Zwighaft et al., 2015). Such variation in polyamine content of photoreceptors has the potential to regulate a variety of processes.

Polyamines are produced naturally from metabolism of L-ornithine, first by decarboxylation to putrescine, followed by sequential N-alkylation reactions with S-adenosylmethionine to form spermidine and spermine. Polyamines have very diverse roles in metabolism, but perhaps some of the most relevant to this study are prominent effects on a number of ion channels (Williams, 1997; Pegg, 2016). The steep, voltage-dependent rectification of inward rectifier potassium channels is caused by channel block by intracellular polyamines (Ficker et al., 1994; Lopatin et al., 1995; Nichols and Lee, 2018). Somewhat similar open channel block also occurs in some kainate and calcium-permeable AMPA receptors (Bowie, 2018), NMDA receptors and a variety of other cation-selective channels (Skatchkov et al., 2014; Pegg, 2018). Among these is the photoreceptor cyclic nucleotide-gated channel (Lu and Ding, 1999); polyamine block of this channel is proposed to suppress noise in the phototransduction cascade.

Intracellular spermine has also been found to efficiently block Connexin 40 (Cx40) gap junctions in a voltage-dependent manner (Musa and Veenstra, 2003), while spermine and spermidine enhance coupling and reduce pH-induced closure of Cx43-containing channels (Skatchkov et al., 2015). Thus, effects on gap junctions cannot be directly predicted; the effects of polyamines on the Cx36 gap junctions present in photoreceptors have not been studied. Finally, polyamines have been found to inhibit the GTPase activity of Gi proteins (Daeffler et al., 1999). The elevated daytime concentration of polyamines in photoreceptor terminals would increase the persistence of Gi signaling driven by dopamine, which would reinforce the potent diurnal gap junction uncoupling observed among photoreceptors of most vertebrate species (Ribelayga and O’Brien, 2017; O’Brien, 2019).

While ion channels regulated by polyamines have not been specifically identified in photoreceptor terminals, several such channels are present postsynaptically. If photoreceptor polyamines were to regulate these channels, it would be necessary for them to be released. There is some evidence that polyamines can be packaged into vesicles with neurotransmitters and be released by neurons (Moriyama et al., 2020). We did not find clear evidence for the recently identified vesicular polyamine transporter SLC18B1 in photoreceptor terminals, but instead found that it is likely to be present in Müller glia. However, it has been shown that the properties of polyamine transporters in brain synaptic vesicles and syanptosomes differ from those in glial cells (Masuko et al., 2003), suggesting that a different, as yet unidentified polyamine transport mechanism may still be present in synaptic vesicles of photoreceptors. Furthermore, the single-cell transcriptome data (Hoang et al., 2020) that we analyzed revealed a low level of SLC18B1 transcript in rods, implying that this transporter could be expressed there. Finally, it has been observed that polyamines can be transported both into and out of cells through a variety of plasma membrane organic cation transporters (Moriyama et al., 2020), providing an alternative mechanism through which polyamines may be released.

Extracellular polyamines can be transported through several types of ionotropic glutamate receptors (Bowie, 2018), pannexins and connexin hemichannels (Skatchkov et al., 2014), potentially providing a path for uptake locally into horizontal cell dendritic tips where Kir2.1 is situated. If such a pathway functions in the retina, it would be unlikely to influence the large population of Kir2.1 covering the horizontal cell soma and proximal dendrites. Extracellular polyamines can also have direct modulatory functions on several ion channels (Moriyama et al., 2020). Extracellular polyamines potentiate Glur6-containing kainate receptors by relieving proton block (Mott et al., 2003). This effect is independent of the open channel block of the pore by intracellular polyamines. Extracellular polyamines also potentiate NMDA receptors at low concentrations (Ogden and Traynelis, 2011). In the photoreceptor synaptic complex, GluR6/7-containing kainate receptors found on the tips of rat, cat, macaque and rabbit horizontal cell processes (Brandstatter et al., 1997; Vardi et al., 1998; Haverkamp et al., 2000, 2001; Vila et al., 2017) could be subject to this type of regulation. We cannot exclude this mechanism from functioning in the mouse, as functional kainate receptors are present in mouse horizontal cells (Schubert et al., 2006; Stroh et al., 2013; Feigenspan and Babai, 2015) and GluR6 transcript is present (Hoang et al., 2020).

Our study found significant polyamine labeling and the presence of the vesicular polyamine transporter SLC18B1 in Müller cells. Müller glia display regional variation in polyamine content that closely correlates to the degree of rectification of inward rectifier potassium currents, with the highest content and greatest rectification at the endfeet (Skatchkov et al., 2000). Our finding of the presence of the vesicular polyamine transporter suggests that Müller cells have the ability to release polyamines into the retina. With many potential targets, the effects of such release on retinal functions may be complex. Indeed, knockout of SLC18B1 in mice has been shown to result in short- and long-term memory deficits (Fredriksson et al., 2019), indicating that vesicular release of polyamines elsewhere in the CNS has important consequences.

Our study in mouse retina reveals that a suite of proteins restricted to the tips of horizontal cell processes is conserved among mammals. Conserved elements of this suite are Chapsyn110, Kir2.1 and likely a kainate receptor. The presence of a suite of proteins narrowly restricted to horizontal cell dendritic and axon terminal tips suggests a function specific to synaptic communication with photoreceptors. Because this suite is localized deep within the synaptic invagination of photoreceptors, we presume that it may function in feedback signaling from horizontal cells to photoreceptors, as well as feedforward signaling from the photoreceptors to horizontal cells. This study makes possible further investigation of the functional significance of this suite in the genetically tractable mouse model.

## Data Availability Statement

The raw data supporting the conclusions of this article will be made available by the authors, without undue reservation.

## Ethics Statement

The animal study was reviewed and approved by the Animal Welfare Committee The University of Texas Health Science Center at Houston.

## Author Contributions

AV planned the research, performed the research, made the figures and wrote the initial draft of the manuscript. ES, ZZ, and AS performed the research. CR provided resources and acquired funding. JO’B directed the research, acquired funding, made figures, and wrote the final draft of the manuscript. All authors reviewed and revised the manuscript.

## Conflict of Interest

The authors declare that the research was conducted in the absence of any commercial or financial relationships that could be construed as a potential conflict of interest.

## Publisher’s Note

All claims expressed in this article are solely those of the authors and do not necessarily represent those of their affiliated organizations, or those of the publisher, the editors and the reviewers. Any product that may be evaluated in this article, or claim that may be made by its manufacturer, is not guaranteed or endorsed by the publisher.

## Footnotes

1
https://proteinpaint.stjude.org/F/2019.retina.scRNA.html
